# Christian Scientists

**Published:** 1898-10-15

**Authors:** 


### The Hospital

A Journal of

The Medical Sciences and Hospital Administration.

Vol. XXV.—No. 629.] Edited by Sir Henry Burdett, K.C.B., and Solomon C. Smith, M.D., M.R.C.P. [Oct. 15, 1898.

### "CHRISTIAN SCIENTISTS."

The newspapers last week reported a melancholy example of folly and superstition, such as could hardly have been deemed possible at this period of the world's history, in relation to the death of an unfortunate gentleman named Lester, a Major in the Prince of Wales's West Yorkshire Regiment, and an Instructor in Military Topography at Sandhurst. It appeared from the evidence given at the inquest, which was held at Camberley before Mr. Roumieu, that the deceased had for some time been suffering from tuberculous peritonitis, and that medical men had practically abandoned any expectation of his recovery. The medical witnesses had only attended him from August 19th to September 5th, and, as the inquest was held on October 10th, presumably within two or three days of the death, they had, perhaps, been a little hasty in giving an unfavourable prognosis. Nothing was said at the inquest about the treatment, further than that morphia was injected for the relief of pain, so that it does not appear whether any operative proceeding had either been advised or had recourse to. But, seemingly as a consequence of the opinion given with regard to the probable issue of the case, the medical attendants were informed on September 5th that their professional services were no longer required, and a certain Mrs. Grant, who describes herself as a "Christian Scientist," was called in. The title seems to demand some explanation. Christianity, as we understand it, is eminently practical; whereas the basis of Mrs. Grant's treatment appears to have been that she should "think about" the sufferer, or at least that she should say that she was thinking about him, an expedient which would probably have answered quite as well, and which, at any rate, could not have failed more completely. "Scientist" is, perhaps, still more puzzling. We have seen the word in type on many occasions, but we do not know either what it means or of what language it forms part. It may possibly be an error for "sciolist," a word which does exist, and which signifies an ignorant pretender to knowledge. However this may be, Mrs. Grant duly thought about the patient, or said that she thought about him, from the time when she took possession of the case until his death, about a month afterwards; and she asserts that he improved under her thinking, and became able to go out. As to this, there was no independent testimony; but it is clear that the alleged improvement could at any rate have been of no more value than was that of the often-mentioned Frenchman who "died cured" as a result of the administration of his physician's favourite remedy.

The practices of "Christian Scientists," and of "Peculiar People," and of faith-healers generally, ought by this time to be perfectly intelligible to all educated persons; and a very good, although too brief, account of the facts on which these practices depend has been given by Dr. Lauder Brunton in his well-known book on the "Action of Medicines." It should be common knowledge that there are many conditions, chiefly depending upon perversions of nervous and vascular action, and the latter probably merely as a consequence of the former, in which suggestion may be made to exert an extremely powerful influence, so as to succeed in banishing symptoms of a particular class, while others, often of some superficial resemblance to the former, are left wholly unaffected. Dr. Lauder Brunton relates the case of a lady who received an injury to one of her legs. She was treated by various surgeons in London, and the injury was recovered from, but she could not be made to believe the assurances of her doctors that she could walk, and she would not try. She went on a pilgrimage to Lourdes, and, after two days, threw away her crutches and walked as well as other people. Cases of this sort present no difficulty to rational folk; but they are constantly seized upon by mountebanks who make a trade of religion, and are exploited from the point of view of the miraculous. No great harm would be done if the practices of such people were confined to appropriate subjects, and no great fuss need be made about such subjects, whether they were healed by faith or remained in their unhealed condition. But when the mountebank intervenes in a case of serious illness, and more especially in illness of a kind which, even in very unpromising examples, has in a considerable proportion of them been found to yield to surgical treatment, the folly of the proceeding must in great measure be concealed beneath its wickedness. The people who offered up their children to Moloch were in no way worse than those who sacrifice human life upon the twin altars of greed and superstition.

